# An intraoperative nomogram for predicting secondary margin positivity in breast conserving surgery utilizing frozen section analysis

**DOI:** 10.3389/fonc.2024.1366467

**Published:** 2025-01-07

**Authors:** Cheng Li, Yan Jiang, Xumiao Wu, Yong Luo, Qi Li

**Affiliations:** ^1^ Department of Breast and Thyroid Surgery, Ningbo Medical Center Lihuili Hospital, Ningbo, Zhejiang, China; ^2^ Department of Ultrasound, Ningbo Medical Center Lihuili Hospital, Ningbo, Zhejiang, China; ^3^ Department of Radiology, Ningbo Medical Center Lihuili Hospital, Ningbo, Zhejiang, China

**Keywords:** breast conserving surgery, frozen section analysis, nomogram predictive model, surgical margin positivity, intraoperative decision making, margin assessment

## Abstract

**Background:**

Breast conserving surgery (BCS) is a standard treatment for breast cancer. Intraoperative frozen section analysis (FSA) is widely used for margin assessment in BCS. In addition, FSA-assisted excisional biopsy is still commonly practiced in many developing countries. The aim of this study is to develop a predictive model applicable to BCS with FSA-assisted excisional biopsy and margin assessment, with a focus on predicting the risk of secondary margin positivity in re-excision procedures following positive initial margins. This may reduce surgical complications and healthcare costs associated with multiple re-excisions and FSAs for recurrent positive margins.

**Methods:**

Patients were selected, divided into training and testing sets, and their data were collected. The Least Absolute Shrinkage and Selection Operator (LASSO) was used to identify significant variables from the training set for model building. Model performance was evaluated using Receiver Operating Characteristic (ROC) curves, calibration curves, and Decision Curve Analyses (DCAs). An optimal threshold identified by the Youden index was validated using sensitivity, specificity, positive predictive value (PPV), and negative predictive value (NPV).

**Results:**

The study included 348 patients (256 in the training set, 92 in the testing set). No significant statistical differences were found between the sets. LASSO identified six variables to construct the model and corresponding nomogram. The model showed good discrimination (mean area under the curve (AUC) values of 0.79 in the training set and 0.83 in the testing set), calibration (Hosmer-Lemeshow test results (*p*-values 0.214 in the training set, 0.167 in testing set)) and clinical utility. The optimal threshold was set at 97 points in the nomogram, yielding a sensitivity of 0.66 (0.54-0.77), specificity of 0.80 (0.74-0.85), PPV of 0.56 (0.47-0.64) and NPV of 0.86 (0.82-0. 90) for the training set, and a sensitivity of 0.65 (0.46-0.84), specificity of 0.88 (0.79-0.95), PPV of 0.68 (0.53-0.85) and NPV of 0.87 (0.81-0.93) for the testing set, demonstrating the model’s effectiveness in both sets.

**Conclusions:**

This study successfully developed a novel predictive model for secondary margin positivity applicable to BCS with FSA-assisted excisional biopsy and margin assessment. It demonstrates good discriminative ability, calibration, and clinical utility.

## 1 Introduction

Breast cancer remains the most commonly diagnosed cancer in women worldwide and is a major contributor to cancer-related deaths. In 2020, it was responsible for approximately 685,000 deaths, accounting for 6.9% of all cancer deaths worldwide (1, 2). Breast conserving surgery (BCS) is a standard treatment for early breast cancer. It is recognized for being less invasive, offering cosmetic benefits, high patient satisfaction, and contributing to improved quality of life (3). Successful BCS is characterized by the achievement of “negative margins” (4), which indicates the absence of cancer cells at the edges of the tissue. When negative margins are achieved, BCS is comparable to mastectomy in terms of local recurrence and survival rates (5, 6), Consequently, the detection of “positive margins,” which indicate residual cancer cells at the tissue margins, after BCS necessitates reoperation (7).

In regions where reoperation is less favored due to various factors, the use of intraoperative frozen section analysis (FSA) for margin assessment in BCS has gained popularity (8). FSA provides high sensitivity and specificity for real-time margin assessment during surgery (9, 10), enabling on-the-spot re-excision of margins when positive margins are identified. This method significantly reduces the incidence of subsequent re-operations (11). Additionally, in many developing countries, including various regions in China, the procedure of conducting FSA-assisted diagnostic excisional biopsy before BCS within the same surgical session is still commonly practiced for economic and traditional reasons (8, 12, 13).

However, an FSA assessment often takes more than 30 minutes and requires the expertise of a trained histotechnologist for sample preparation and an experienced pathologist for interpretation (14). If FSA identifies positive margins during BCS, additional, sometimes multiple, re-excisions and subsequent FSAs may be required. This can result in prolonged surgery, longer anesthesia times, and increased operating room occupancy. Together, these factors can increase the risk of surgical complications and lead to higher healthcare costs. To reduce the incidence of recurrent positive margins in BCS with FSA-assisted excisional biopsy and margin assessment, and thereby reduce the frequency of multiple re-excisions and FSAs, predicting the risk of recurrent positive margins is critical. By identifying high risk cases, we can implement preemptive measures, such as increasing margin width, to reduce the likelihood of margin recurrence.

Existing models for assessing the risk of positive margins in BCS primarily rely on preoperative paraffin pathology and/or immunohistochemistry data (15–17). However, these data are often not available for BCS with FSA-assisted excisional biopsy and margin assessment. Therefore, it is essential to explore how to effectively utilize pathological information obtained by FSA for predictive purposes. In addition, these models generally focus on predicting initial margin positivity (16, 18–20). However, for BCS with FSA-assisted excisional biopsy and margin assessment, initial surgical margins are typically assessed at the time of tumor biopsy prior to BCS. Therefore, in this specific workflow, it is becoming increasingly important to predict the risk of positive margins at secondary margin excision in BCS.

The primary objective of this study is to develop a predictive model focused on predicting the risk of secondary margin positivity in re-excision procedures following the detection of initial positive margins. This model incorporates pathology data that can be obtained through intraoperative FSA along with clinical data that can be collected preoperatively. Therefore, the applicability of the model extends to BCS with FSA-assisted excisional biopsy and margin assessment within the same surgical session.

## 2 Methods

### 2.1 Study design and data collection

This study adheres to the principles of the Declaration of Helsinki. Ethical approval (approval number KY2020PJ048) was granted by the Ethics Committee of Ningbo Medical Center Lihuili Hospital, and informed consent was obtained from all participants for both participation and publication. A retrospective analysis was conducted on patients admitted for breast cancer surgery at the Breast Surgery Department of Ningbo Medical Center Lihuili Hospital (Eastern District) from January 1, 2016 to October 1, 2023.

The institution follows to the National Comprehensive Cancer Network’s guidelines for BCS. Our routine preoperative imaging protocol includes both ultrasound and mammography. MRI is used as a supplementary examination when the results of mammography and ultrasound are inconclusive. In the diagnosis of suspicious breast lesions, core needle biopsy represents the primary modality. However, for patients with a medium to high suspicion of malignancy [classified as 4B-5 by imaging according to the American College of Radiology (ACR) Breast Imaging Reporting and Data System (BI-RADS)], and clinical stages T1-T2, N0-N1, M0, excisional biopsy and frozen section analysis will be considered as an alternative to preoperative core needle biopsy if the patient does not prefer this procedure. In situations where the lesion qualifies for BCS during excisional biopsies, we routinely preserve a 1 cm width of normal tissue around the lesions and orient the margins with surgical sutures. This facilitates further examination of the margins if malignancy is confirmed. If FSA or paraffin pathology reports indicate positive margins—characterized by the presence of atypical hyperplasia (including atypical ductal or lobular hyperplasia), *in situ*, or invasive carcinoma at the margin—additional margin excision and a secondary FSA are performed. If positive margins persist, or if additional re-excision may compromise cosmetic outcome, total mastectomy is considered. All surgical procedures are performed by one of the six breast surgeons in our department.

Patients were selected from the institutional database based on specific inclusion and exclusion criteria. Inclusion criteria included: 1) clinical tumor stage cT 1-2, clinical lymph node stage N 0-1, M 0; 2) definitive malignant pathology results from intraoperative FSA or postoperative paraffin pathology; 3) initial BCS; 4) positive initial margin examination followed by secondary FSA-assisted margin examination. Exclusion criteria were: 1) male patients; 2) incomplete imaging, clinical, or pathologic data; 3) history of neoadjuvant chemotherapy; 4) pathologically confirmed multicentric breast cancer; 5) history of previous breast cancer; 6) absence of secondary margin examination or direct conversion to mastectomy after positive initial margins.

Clinical, imaging, and pathologic data were systematically collected for patients who met the inclusion criteria. Images were reviewed independently by two sonographers and two radiologists. All reviewers used a uniform standard to classify the imaging characteristics of the tumors. In cases of disagreement, a senior sonographer or radiologist was consulted for resolution. Importantly, all reviewers were blinded to the pathology reports to ensure unbiased assessments. Ultrasound calcifications were categorized into three groups: no calcification, microcalcification (< 0.5 mm in diameter), and macrocalcification (≥ 0.5 mm) (21). Mammographic calcifications were classified as “suspicious” if the morphology of the calcifications appeared as amorphous, coarsely heterogeneous, fine pleomorphic, fine linear, or fine linear branching (22). Ultrasound axillary lymph nodes were classified as “suspicious” if the sonographic features of the lymph nodes showed cortical thickening, a long-to-short axis ratio < 2, effacement or replacement of the fat hilum, and/or nonhilar blood flow (23). Mammographic breast density was categorized as non-dense (BI-RADS categories A and B) and dense (categories C and D) (22). The classification of other variables was consistent with the descriptors of the ACR BI-RADS, 5*^th^* edition (24). The largest tumor diameter was documented in both ultrasound, mammography, and pathology. If the lesion was not visible on mammography, its mammographic size was recorded as “0”. The pathological breast cancer classification was according to the World Health Organization classification of tumors of the breast, 5*^th^* edition (25). The clinical lymph node stage was determined according to the American Joint Committee Cancer Staging, 8*^th^* edition (26). Positive margins were defined by the presence of atypical hyperplasia, including atypical ductal or lobular hyperplasia, and *in situ* and invasive carcinoma at the margin (27). Patient age and body mass index (BMI) were obtained from medical records, with BMI calculated as weight in kilograms divided by height in meters squared. Clinical evaluation of suspicious axillary lymph nodes was based on physical examination records.

### 2.2 Model construction

Patients admitted between January 1, 2016, and December 31, 2021, formed the training set for our predictive model, while patients admitted between January 1, 2022, and October 1, 2023, formed the testing set. In the training set, we set secondary margin status as the dependent variable. To identify the most predictive variables for this dependent variable, we used Least Absolute Shrinkage and Selection Operator (LASSO) regression. LASSO regression applies a penalty coefficient, λ, to incrementally reduce the coefficients of the independent variables, specifically reducing those with minimal predictive power to zero first (28). This method is recognized for its ability to minimize prediction error and mitigate overfitting (29). Due to a notable correlation between age and breast density, age was removed from the list of independent variables. We performed 10-fold cross-validation to assess the performance of the model across different λ values, using binomial deviance as the criterion for performance evaluation. The optimal λ that produced the lowest binomial deviance was selected. Variables that retained non-zero coefficients at this optimal λ were considered significant and subsequently included in the logistic regression predictive model. For practical use, the model was visualized as a nomogram (30).

### 2.3 Model validation

The discriminative power, calibration, and clinical utility of our model were evaluated in both the training and testing sets. Discriminative power was assessed using receiver operating characteristic (ROC) curves and corresponding area under the curve (AUC) values. Calibration, defined as the agreement between predicted probabilities and actual outcomes, was assessed using calibration curves (31) and the Hosmer-Lemeshow test. A calibration curve that is close to the ideal curve and a larger *p*-value in the Hosmer-Lemeshow test indicate better calibration. Clinical utility was assessed using Decision Curve Analysis (DCA) (32), which compares the net benefit of the model to the two extremes of “intervention for all” or “intervention for none”. Net benefit is calculated from the model’s effectiveness in accurately identifying true positives while reducing false positives. Good clinical utility is indicated when the model’s DCA curve shows a higher net benefit than the “intervention for all” and “intervention for none” at a given probability threshold.

We identified the optimal threshold for distinguishing between low and high risk of positive secondary margins based on the maximum value of the Youden index (sensitivity + specificity - 1) in the training set. To evaluate the performance of the model at this threshold, confusion matrices were constructed for both sets, allowing the calculation of sensitivity, specificity, positive predictive value (PPV), and negative predictive value (NPV).

### 2.4 Statistical analysis

In this study, continuous variables were reported as mean ± standard deviation (SD), while categorical variables were reported as frequencies and percentages. We performed statistical analyzes using R software (version 4.3.1). A variety of R packages facilitated our data processing, modeling, and validation efforts. The “tidyverse” package (33) was used for data manipulation, and the “glmnet” package (34) was used to fit the LASSO regression models. The “rms” package (35) was used for regression modeling and validation, and the “pROC” package (36) was used for ROC curve analysis. The “CompareGroup” package (37) was used for comparative statistical analysis, and the “ggplot2” package (38) was used to generate graphical representations, including plots and graphs. The “rmda” package (39) was used for DCA, and the “ResourceSelection” package (40) was used for the Hosmer-Lemeshow test. To robustly estimate the means and their 95% confidence intervals (CI) for the ROC curve, AUC values, and DCA curve, we used the bootstrap method with 2,000 replications, according to the methods described by Efron (41). A *p*-value < 0.05 was considered statistically significant.

## 3 Results

**Figure 1:**
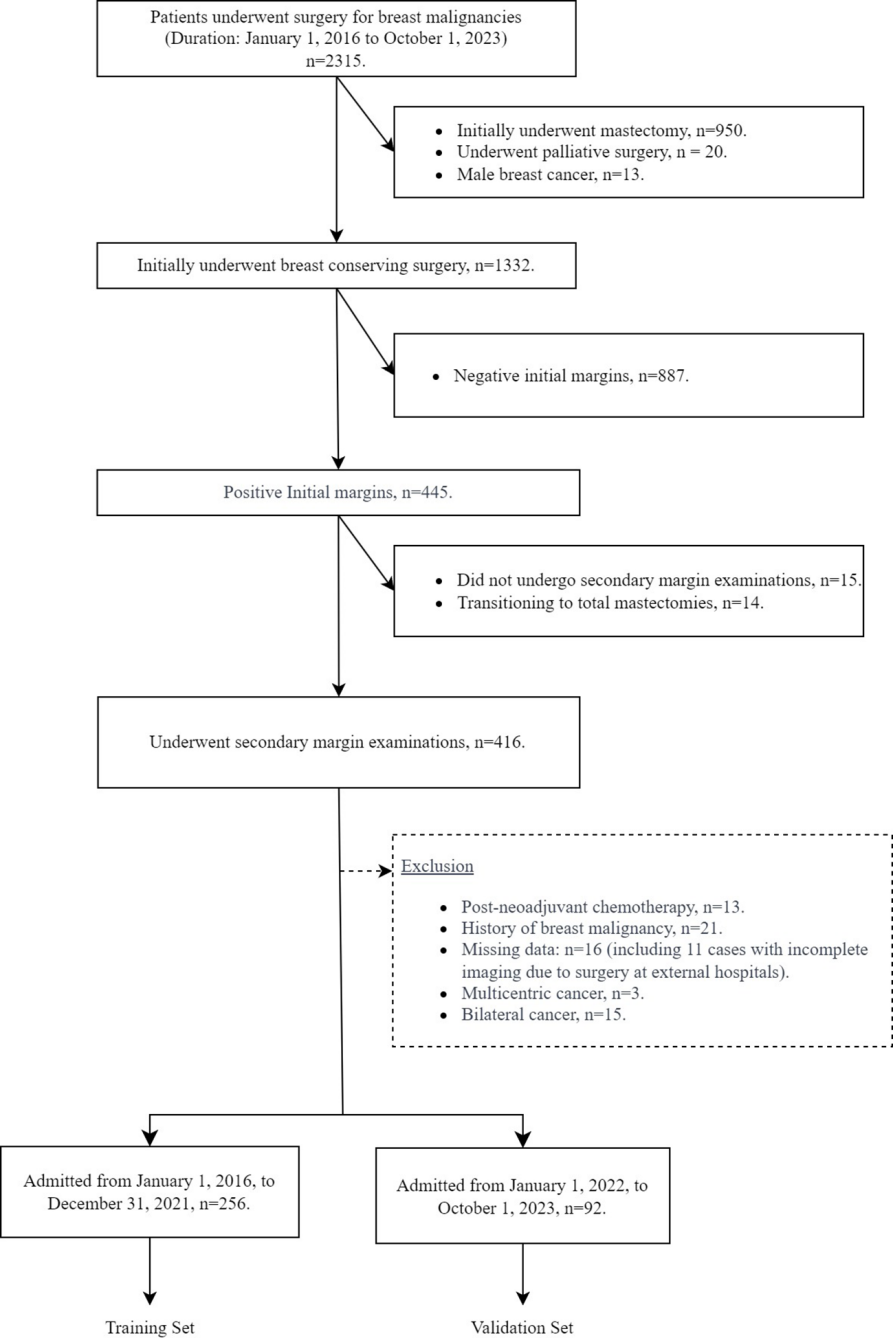
Flow chart of patient inclusion and exclusion.

The patient selection process, including inclusion and exclusion, for this study is shown in **Figure 1**. From January 1, 2016 to October 1, 2023, a total of 2,315 patients underwent breast cancer surgery in our department. Among them, 1,332 women initially underwent BCS for breast cancer. After applying our inclusion and exclusion criteria, 348 patients were identified who had a positive initial margin examination and subsequently underwent a secondary margin examination for study inclusion. Of these patients, 96 had positive secondary margins and 252 had negative secondary margins. The study cohort was divided into two groups for analysis: the training set, consisting of 256 patients admitted from January 1, 2016 to December 31, 2021, and the testing set, consisting of 92 patients admitted from January 1, 2022 to October 1, 2023. As shown in **Table 1**, an analysis of variable distributions and comparative differences within the training and testing sets revealed no statistically significant differences in baseline characteristics between the sets (all *p*-values *>* 0.05), indicating a homogeneous distribution of variables across both sets.

**Table 1:** Comparative analysis of clinicopathological variable distributions between training and testing sets.

Clinicopathological Variable	All N=348	Training chort N=256	Testing chort N=92	*p*
Pathological tumor type, n (%)				0.50
NST	255 (73.3%)	191 (74.6%)	64 (69.6%)	
DCIS	60 (17.2%)	44 (17.2%)	16 (17.4%)	
ILC	15 (4.3%)	9 (3.5%)	6 (6.5%)	
Other Special Invasive Types	18 (5.2%)	12 (4.7%)	6 (6.5%)	
Pathological tumor size (mm), mean ± SD	18.3 ± 8.6	18.2 ± 8.4	18.3 ± 9.0	0.99
Pathological multifocal carcinoma^a^, n (%)				0.79
Absent	330 (94.8%)	242 (94.5%)	88 (95.7%)	
Present	18 (5.2%)	14 (5.5%)	4 (4.3%)	
Initial positive margin count^b^, n (%)				0.50
1	238 (68.4%)	172 (67.2%)	66 (71.7%)	
>1	110 (31.6%)	84 (32.8%)	26 (28.3%)	
Secondary margin examination^c^, n(%)				0.97
Positive	96 (27.6%)	70 (27.3%)	26 (28.3%)	
Negative	252 (72.4%)	186 (72.7%)	66 (71.7%)	
Age (years), mean ± SD	53.4 ± 13.0	53.6 ± 12.9	52.9 ± 13.3	0.66
BMI (kg*/*m^2^), mean ± SD	23.5 ± 3.4	23.4 ± 3.3	23.6 ± 3.7	0.66
cN^d^ Stage, n (%)				0.81
cN0	302 (86.8%)	221 (86.3%)	81 (88.0%)	
cN1	46 (13.2%)	35 (13.7%)	11 (12.0%)	
Mammographic breast density, n (%)				0.14
Non-dense^e^	102 (29.3%)	69 (27.0%)	33 (35.9%)	
Dense^f^	246 (70.7%)	187 (73.0%)	59 (64.1%)	
Mammographic mass shape,n(%)				0.66
Round	46 (13.2%)	32 (12.5%)	14 (15.2%)	
Oval	90 (25.9%)	63 (24.6%)	27 (29.3%)	
Irregular	89 (25.6%)	68 (26.6%)	21 (22.8%)	
Not clearly visible^g^	123 (35.3%)	93 (36.3%)	30 (32.6%)	
Mammographic obscured mass margin, n(%)				0.52
Absent	223 (64.1%)	161 (62.9%)	62 (67.4%)	
Present	125 (35.9%)	95 (37.1%)	30 (32.6%)	
Mammographic suspicious calcifications^h^, n(%)				0.48
Absent	222 (63.8%)	160 (62.5%)	62 (67.4%)	
Present	126 (36.2%)	96 (37.5%)	30 (32.6%)	
Mammographic tumor size(mm), mean ± SD	15.8 ± 11.9	15.6 ± 11.9	16.2 ± 11.7	0.67
Ultrasound mass orientation, n(%)				0.95
parallel	228 (65.5%)	167 (65.2%)	61 (66.3%)	
not parallel	120 (34.5%)	89 (34.8%)	31 (33.7%)	
Ultrasound mass shape, n(%)				0.90
Round	53 (15.2%)	39 (15.2%)	14 (15.2%)	
Oval	179 (51.4%)	130 (50.8%)	49 (53.3%)	
Irregular	116 (33.3%)	87 (34.0%)	29 (31.5%)	
Ultrasound mass margins, n(%)				0.43
Circumscribed	21 (6.0%)	16 (6.2%)	5 (5.4%)	
Indistinct	32 (9.2%)	26 (10.2%)	6 (6.5%)	
Microlobulated	141 (40.5%)	102 (39.8%)	39 (42.4%)	
Spiculated	31 (8.9%)	19 (7.4%)	12 (13.0%)	
Angular	123 (35.3%)	93 (36.3%)	30 (32.6%)	
Ultrasound mass echo characteristics, n(%)				0.90
Isoechoic	1 (0.3%)	1 (0.4%)	0 (0.00%)	
Hypoechoic	267 (76.7%)	198 (77.3%)	69 (75.0%)	
Complex cystic and solid	29 (8.3%)	20 (7.8%)	9 (9.8%)	
anechoic	14 (4.0%)	11 (4.3%)	3 (3.3%)	
Heterogenous	37 (10.6%)	26 (10.2%)	11 (12.0%)	
Ultrasound calcification type, n(%)				0.78
Macrocalcification	6 (1.7%)	5 (2.0%)	1 (1.1%)	
Microcalcification	182 (52.3%)	136 (53.1%)	46 (50.0%)	
No calcification	160 (46.0%)	115 (44.9%)	45 (48.9%)	
Ultrasound mass posterior features, n(%)				0.66
Shadowing	52 (14.9%)	37 (14.5%)	15 (16.3%)	
Enhancement	23 (6.6%)	17 (6.6%)	6 (6.5%)	
Combined pattern	25 (7.2%)	16 (6.3%)	9 (9.8%)	
No posterior features	248 (71.3%)	186 (72.7%)	62 (67.4%)	
Ultrasound vascularity, n(%)				0.76
Absent	140 (40.2%)	102 (39.8%)	38 (41.3%)	
Vessels in rim	29 (8.3%)	23 (9.0%)	6 (6.5%)	
Internal vascularity	179 (51.4%)	131 (51.2%)	48 (52.2%)	
Ultrasound architectural distortion, n(%)				0.17
Absent	331 (95.1%)	246 (96.1%)	85 (92.4%)	
Present	17 (4.9%)	10 (3.9%)	7 (7.6%)	
Ultrasound ductal changes, n(%)				0.98
Absent	327 (94.0%)	240 (93.8%)	87 (94.6%)	
Present	21 (6.0%)	16 (6.2%)	5 (5.4%)	
Ultrasound suspicious axillary lymph nodes^i^, n(%)				0.87
Absent	299 (85.9%)	219 (85.5%)	80 (87.0%)	
Present	49 (14.1%)	37 (14.5%)	12 (13.0%)	
Ultrasound tumor size(mm), mean ± SD	18.8 ± 8.77	18.8 ± 8.95	18.7 ± 8.30	0.90
Size difference: pathology vs mammogram(mm), mean ± SD	2.49 ± 10.2	2.62 ± 10.1	2.12 ± 10.4	0.69
Size difference: pathology vs ultrasound(mm), mean ± SD	-3.01 ± 10.9	-3.20 ± 11.2	-2.48 ± 10.1	0.57
Size difference: mammogram vs ultrasound(mm), mean ± SD	-0.53 ± 6.58	-0.60 ± 6.64	-0.36 ± 6.43	0.76

NST, Invasive Carcinoma of No Specific Type; DCIS, Ductal Carcinoma in Situ; ILC, Invasive Lobular Carcinoma; BMI,Body Mass Index.

a Defined as the presence of multiple tumor foci within a single breast quadrant.

b Refers to the number of positive margins identified during initial margin examination in breast conserving surgery. A positive margin is characterized by the detection of atypical hyperplasia (including atypical cells, atypical ductal hyperplasia, and atypical lobular hyperplasia) or the presence of *in situ* and invasive carcinoma at the surgical margin.

c Defined as the performance of an additional margin excision and frozen section analysis following the detection of positive results in the initial margin examination.

d Represents “clinical lymph node stage” classified according to the criteria of the 8*^th^* edition of the American Joint Committee on Cancer Staging.

e Represents breast density categories A and B, as defined by the American College of Radiology (ACR)’s Breast Imaging Reporting and Data System (BI-RADS), 5*^th^* edition.

f Represents breast density categories C and D, as defined by the ACR BI-RADS 5*^th^* edition.

g Attributes to factors such as high breast density that can obscure mammographic details.

h Mammographic calcifications characterized as amorphous, coarse heterogeneous, fine pleomorphic, fine linear, or fine-linear branching

i Axillary lymph nodes on ultrasound are characterized by cortical thickening, a round shape with a long to short axis ratio < 2, effacement or obliteration of the fatty hilum, and/or blood flow patterns not centered in the hilum.

**Figure 2:**
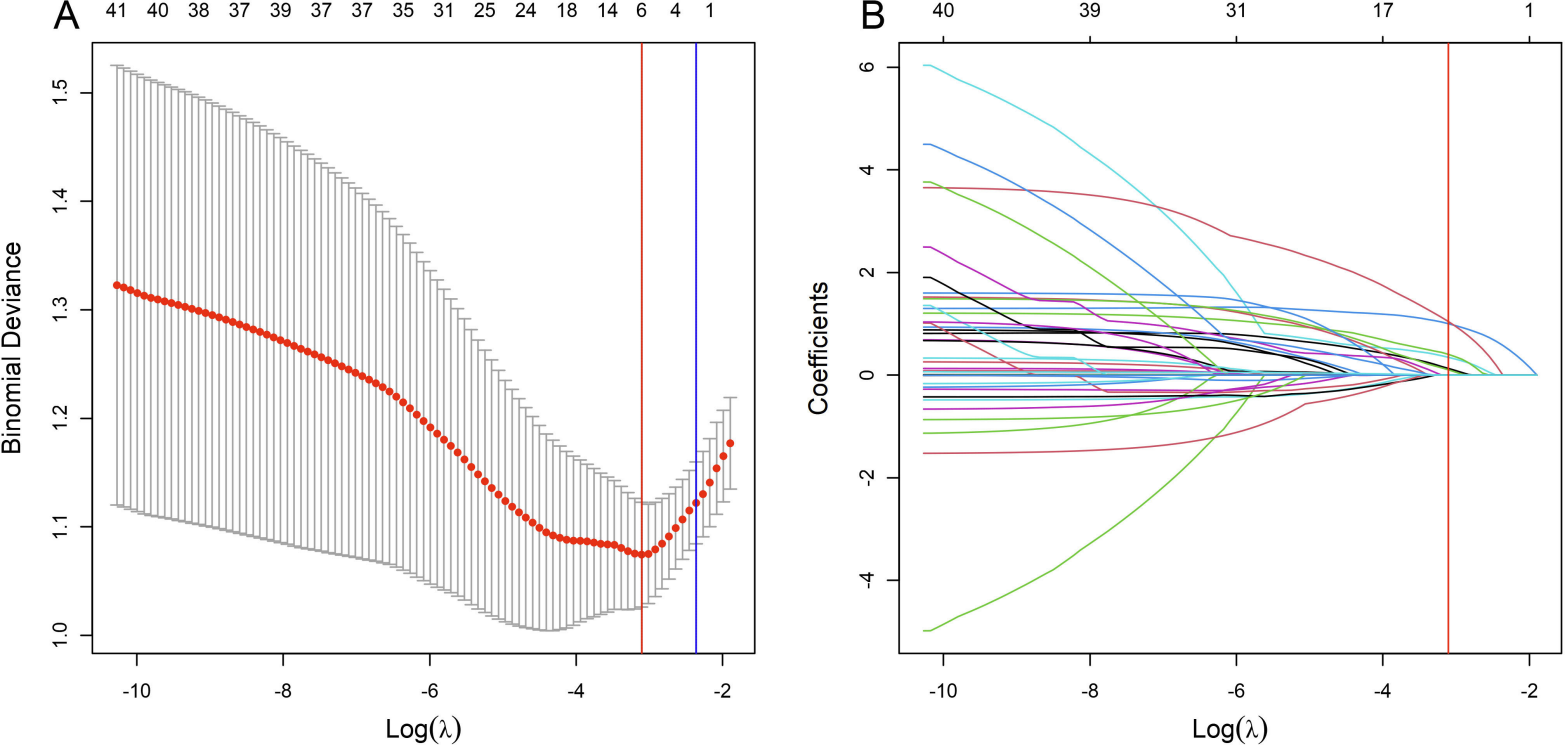
Variable selection process using the Least Absolute Shrinkage and Selection Operator (LASSO) algorithm in the training set. **(A)** shows the LASSO cross-validation curve of the binomial deviance, a measure of model fit, plotted against the log-transformed penalty parameter, log λ. The mean binomial deviance is indicated by red dots, and its standard error by the surrounding error bars, for various values of log λ. At the top of the plot, the enumerated non-zero coefficients for the corresponding log λ values indicate the number of significant predictors retained during regularization. **(B)** shows the progression of the coefficients of the predictors within the LASSO as log λ varies. Individual paths are marked by unique colors, each representing the change in coefficient magnitude as log λ increases. The blue vertical line marks the log λ within one standard error of the minimum deviation, while the red vertical line indicates the log λ associated with the model’s minimum deviation. Predictors with non-zero coefficients at the log λ of the red line are identified for inclusion in the final logistic regression model.

**Figure 3:**
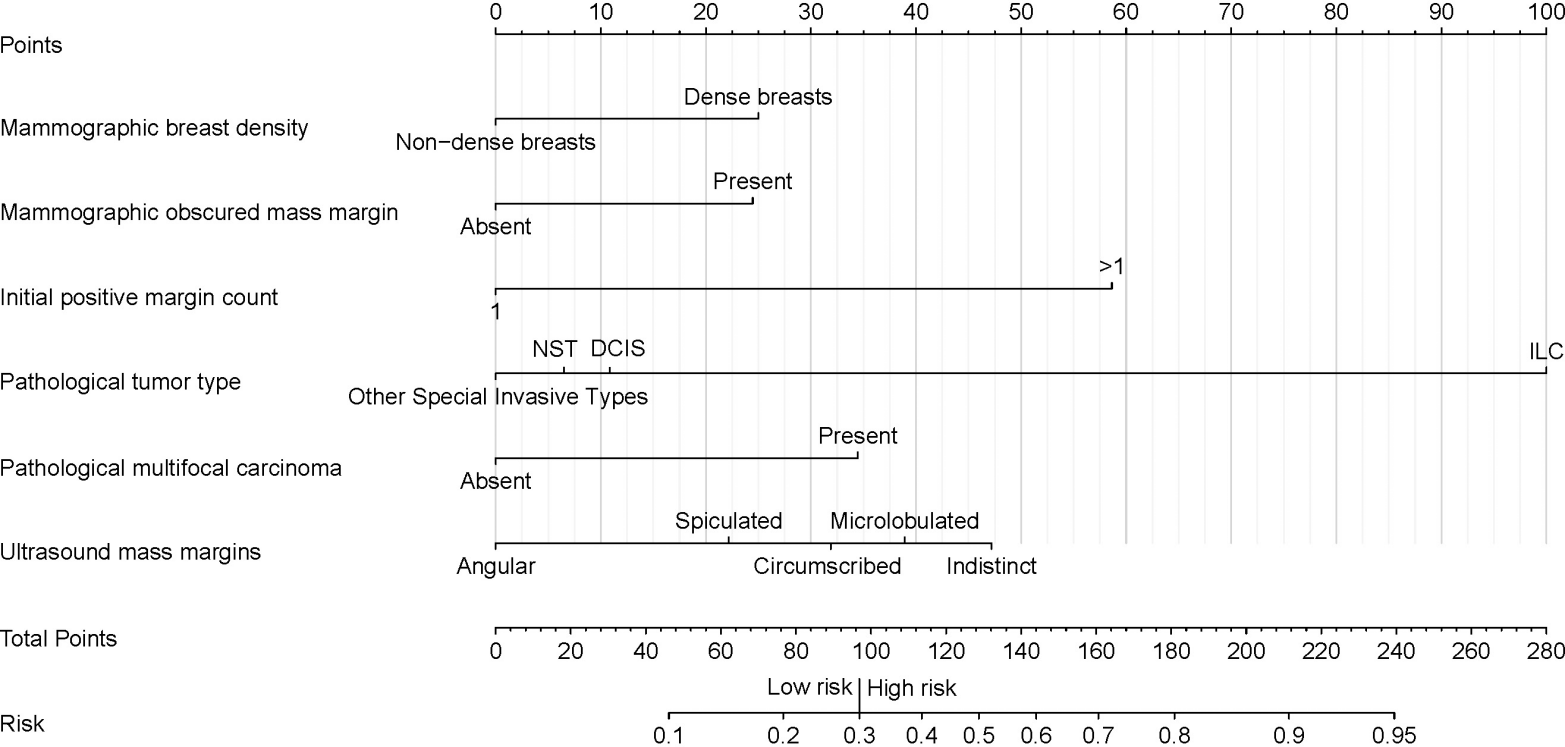
Nomogram for predicting the risk of secondary margin positivity in re-excision procedures after initial margin positivity in breast conserving surgery. Assign risk points to each variable by aligning them with the corresponding position on the “Points scale, then sum these points to determine the overall risk score, which is located on the “Total Points” axis. This score translates directly to a risk percentage on the “Risk” axis, which indicates the likelihood of secondary margin positivity during re-excision after initial positive margins in breast conserving surgery. In this nomogram, a total score of 97 (corresponding to a risk of 0.3) serves as the threshold for distinguishing between low and high risk of secondary margin positivity. Non-dense breast' represents breast density categories A and B, and 'Dense breasts' represents breast density categories C and D, as defined by the ACR BI-RADS 5th edition. “Initial positive margin count” represents the number of positive margins identified during initial margin examination in breast-conserving surgery. A positive margin is characterized by the detection of atypical hyperplasia (including atypical cells, atypical ductal hyperplasia, and atypical lobular hyperplasia) or the presence of *in situ* and invasive carcinoma at the surgical margin. NST, Invasive Carcinoma of No Special Type; DCIS, Ductal Carcinoma *In Situ*; ILC, Invasive Lobular Carcinoma.

During cross-validation of the LASSO over a range of λ values, the optimal λ was found to be 0.0447 (*log*λ = -3.1), corresponding to the point where the binomial deviation reached its minimum, as shown in **Figure 2**. At this specific λ value, six variables with non-zero coefficients were retained, as detailed in **Figure 2**. These variables, namely mammographic breast density, obscured mammographic mass margin, initial positive margin count, pathological tumor type, pathological multifocal carcinoma, and ultrasound mass margins, were subsequently included in the final logistic regression predictive model, as shown in **Table 2**. The model demonstrates significant predictive ability, as indicated by a chi-squared statistic (χ^2^) of 61.09 and a *p*-value < 0.01. In addition, its pseudo-R^2^ value of 0.31 supports this assessment. For practical application, a nomogram based on this logistic regression model has been constructed as shown in **Figure 3**.

**Table 2:** Coefficients and corresponding odds ratios for secondary margin positivity for each variable level in the logistic model.

Clinicopathological variables	Coefficients		Odds Ratios		*p*
	Mean	95%CI	Mean	95%CI	
Mammographic breast density					
Non-dense^a^	Ref.		Ref.		
Dense^b^	0.66	-0.19-1.52	1.94	0.83-4.58	0.13
Mammographic suspicious calcifications^c^					
Absent	Ref.		Ref.		
Present	0.65	-0.03-1.34	1.92	0.97-3.80	0.06
Initial Positive Margin Count					
1	Ref.		Ref.		
>1	1.56	0.90-2.22	4.76	2.46-9.21	<0.01
Pathological tumor type					
Other special invasive types	Ref.		Ref.		
NST	0.17	-1.37-1.71	1.19	0.25-5.55	0.83
DCIS	0.29	-1.38-1.95	1.34	0.25-7.06	0.73
ILC	2.66	0.35-4.97	14.29	1.42-143.65	0.02
Pathological multifocal carcinoma^d^					
Absent	Ref.		Ref.		
Present	0.92	-0.35-2.19	2.5	0.70-8.91	0.16
Ultrasound mass margins					
Angular	Ref.		Ref.		
Circumscribed	1.25	0.13-2.38	2.33	0.63-8.61	0.20
Indistinct	1.03	0.25-1.82	3.51	1.14-10.81	0.03
Microlobulated	0.85	-0.46-2.15	2.81	1.28-6.18	0.01
Spiculated	0.59	-0.78-1.96	1.80	0.46-7.11	0.40

χ^2^ (11) = 61.09, p<0.01

Ref., Reference category; NST, Invasive carcinoma of no special Ttype; DCIS, ductal carcinoma *in situ*; ILC, Invasive lobular carcinoma. CI, Confident interval.

a Denotes the breast density categories A and B as defined in the 5*^th^* edition of the American College of Radiology (ACR) Breast Imaging Reporting and Data System (BI-RADS).

b Denotes the breast density categories C and D as defined in the 5*^th^* edition of ACR BI-RADS.

c Refers to calcifications on mammography appearing as amorphous, coarse heterogeneous, fine pleomorphic, fine linear, or fine-linear branching types.

d Definition as the presence of two or more tumor foci in one breast quadrant.

**Figure 4:**
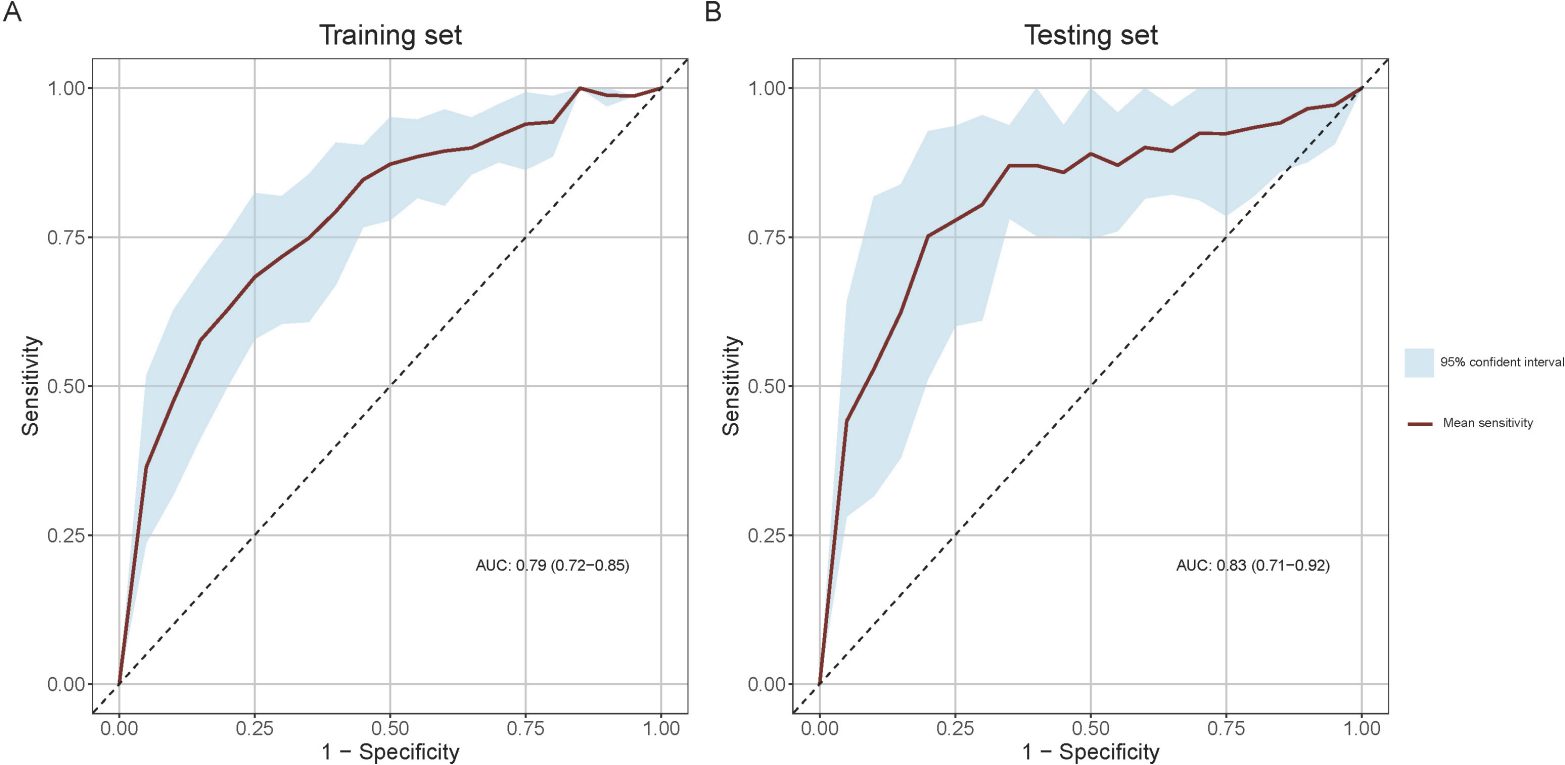
Receiver Operating Characteristic (ROC) curves for model performance evaluation. **(A)** ROC curve in the training set and **(B)** ROC curve in the testing set. The light blue shaded areas delineate the 95% confidence intervals for the mean ROC curves, derived from bootstrap sampling methods.

The mean ROC curves of the model for both training and testing sets, along with their 95% CI bands, are shown in **Figure 4**. The training set achieved a mean AUC of 0.79 (0.72-0.85), while the testing set achieved a mean AUC of 0.83 (0.71-0.92), indicating high discriminative ability.

In the training set, the model achieved its maximum Youden index of 0.45 at a threshold of 0.3, corresponding to a nomogram score of 97 points, which was adopted as the optimal threshold for discriminating between high and low risk of secondary margin positivity. The confusion matrix and performance metrics for this threshold are detailed in **Table 3**. In the training set, the sensitivity, specificity, PPV, and NPV were 0.66 (0.54-0.77), 0.80 (0.74-0.85), 0. 56 (0.47-0.64), and 0.86 (0.82-0.90), respectively, while in the testing set these metrics were 0.65 (0.46-0.84), 0.88 (0.79-0.95), 0.68 (0.53-0.85), and 0.87 (0.81-0.93), respectively. These results indicate the model’s robust predictive accuracy in both sets.

**Table 3:** Confusion matrix and performance metrics of the predictive model for pathological secondary margin status in training and testing sets.

Pathological result Model predicted		Positive	Negative	Sensitivity		Specificity		PPV		NPV	
				Mean	95%CI	Mean	95%CI	Mean	95%CI	Mean	95%CI
Training set				0.66	0.54-0.77	0.80	0.74-0.85	0.56	0.47-0.64	0.86	0.82-0.90
High risk^a^, n(%)		46(65.7)	37(19.9)								
Low risk^b^, n(%)		24(34.3)	149(80.1)								
Testing set				0.65	0.46-0.84	0.88	0.79-0.95	0.68	0.53-0.85	0.87	0.81-0.93
High risk^a^, n(%)		17(65.4)	8(12.1)								
Low risk^b^, n(%)		9(34.6)	58(87.9)								

On the left side of the table is the confusion matrix, which shows the actual positive and negative surgical margins as confirmed by pathology, along with the positive and negative margins predicted by the model. On the right side, the sensitivity, specificity, PPV and NPV are calculated based on the data from the confusion matrices in both the training and testing sets.

PPV, Positive predictive value; NPV, Negative predictive value; CI, Confident interval.

a Model predict risk ≥ 0.3, or nomogram score ≥ 97 points.

b Model predict risk < 0.3, or nomogam score < 97 points.

**Figure 5:**
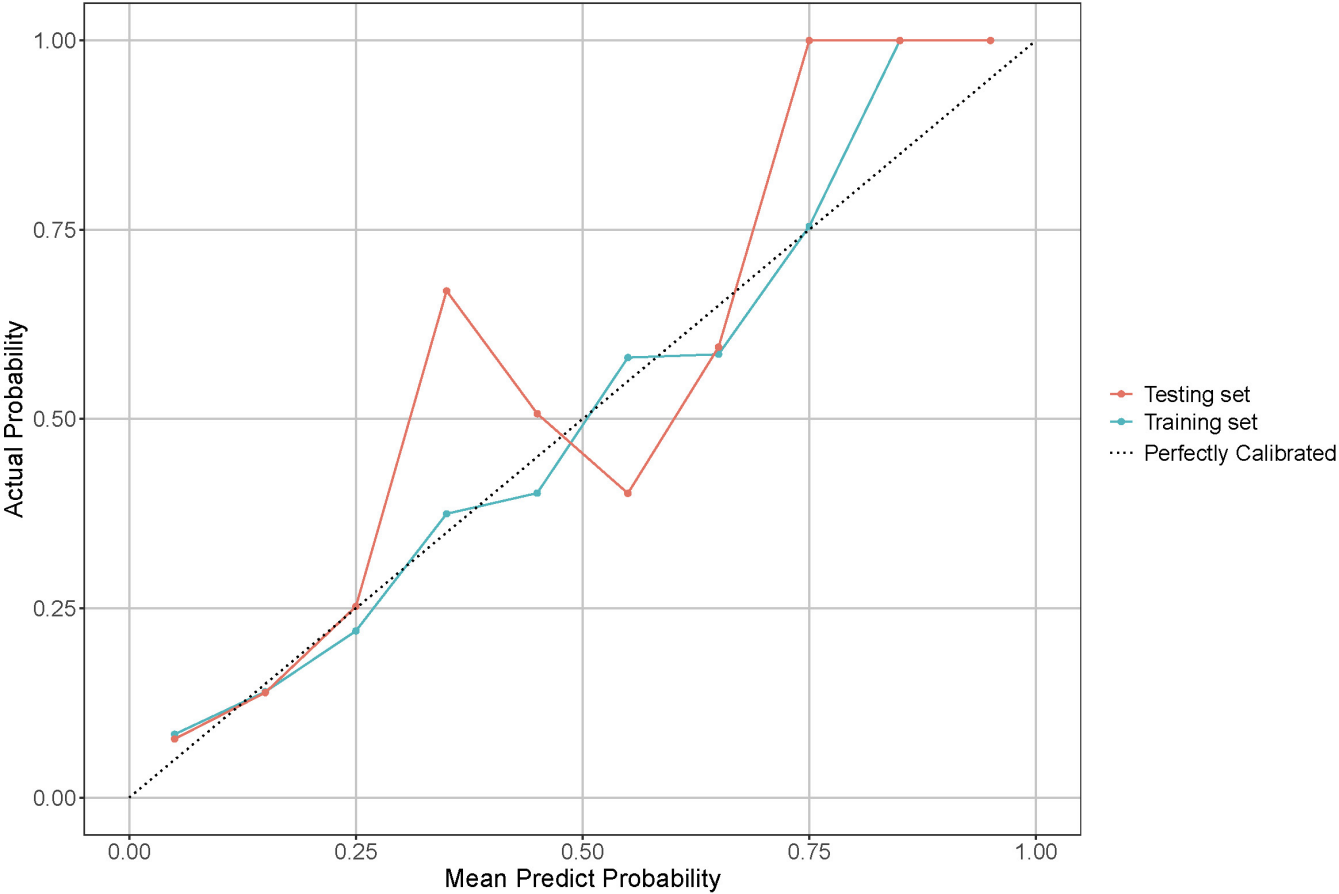
Calibration curve for model performance evaluation in training and testing sets. The dotted line indicates a perfectly calibrated model, where the predicted probabilities exactly match the actual probabilities. The closer the model’s calibration line is to the dotted line, the better the model is calibrated.

The calibration curves for the model in both the training and testing sets are shown in **Figure 5** and demonstrate a close match between the predicted probabilities and the actual results. The Hosmer-Lemeshow test results support this, with a *p*-value of 0.214 (0.204-0.224) for the training set and 0.167 (0.158-0.176) for the testing set, indicating good calibration.

**Figure 6:**
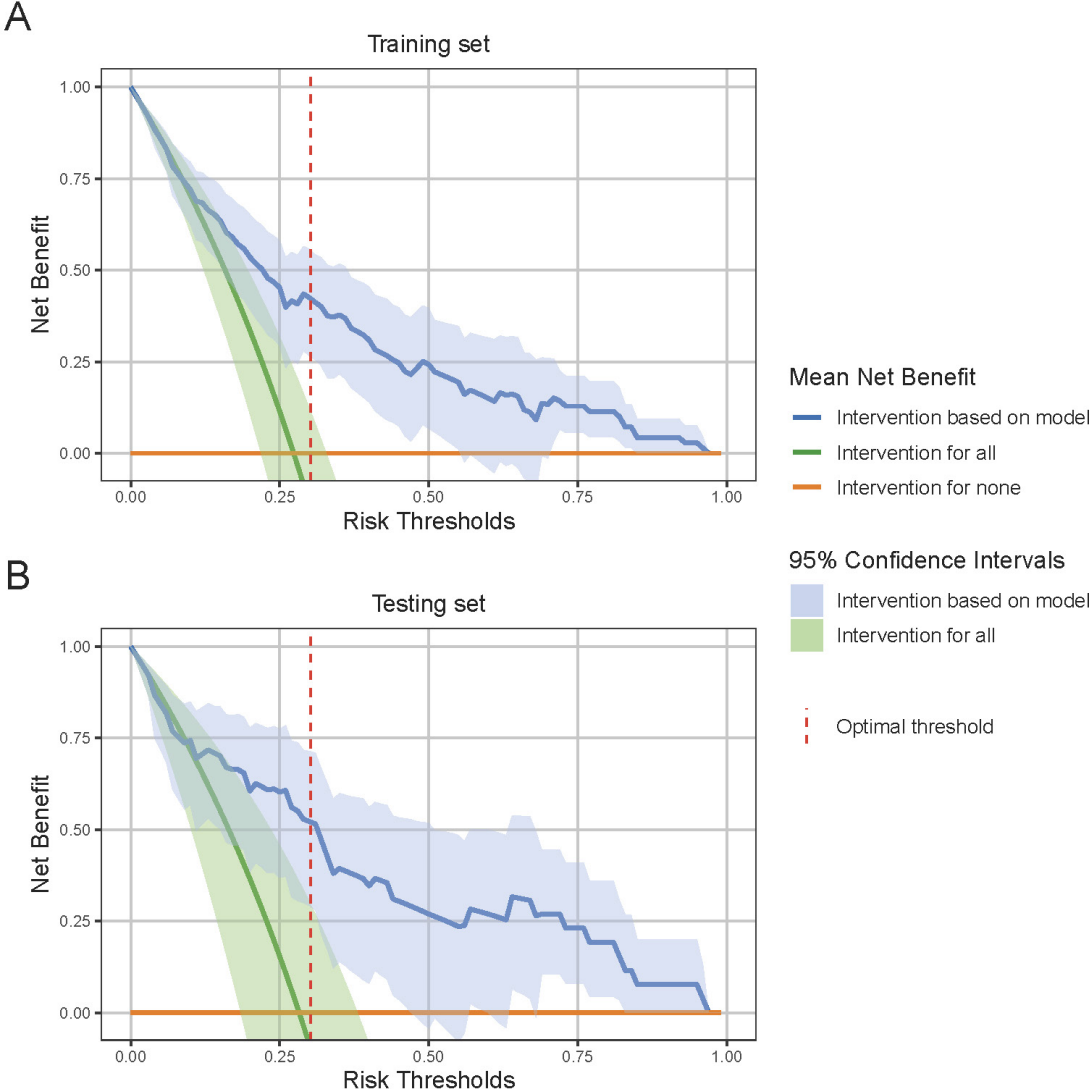
Decision Curve Analysis (DCA) for model performance evaluation. **(A)** DCA curve in the training set and **(B)** DCA curve in the testing set. DCA evaluates the clinical utility of the model by comparing the net benefit of model-based interventions to strategies of intervention for all or none. The 95% confidence interval shade area are derived from bootstrap sampling methods. At the optimal risk threshold, indicated by the red dashed line, the model shows a significantly higher net benefit compared to the “intervention for all” and “intervention for none” strategies, indicating robust clinical utility.

Finally, the DCA for both the training and testing sets is illustrated in **Figure 6**, which shows the mean DCA curves along with their 95% CI bands. Notably, the mean net benefit of the model consistently exceeds that of the “intervention for all” and “intervention for none” strategies across a range of threshold probabilities. This is particularly evident at the optimal threshold, where the model’s mean net benefit and its 95% CI are significantly higher than these strategies in both sets. Such results highlight the model’s potential to improve patient outcomes, providing robust evidence for its clinical utility.

## 4 Discussion

This study successfully developed a novel predictive model for secondary margin positivity applicable to BCS with FSA-assisted excisional biopsy and margin assessment.

During BCS, when the initial margin FSA result is positive, surgeons typically perform a margin re-excision and re-submitte for FSA within the same operative session. At this critical juncture, the surgeon must decide whether to perform a conservative re-excision with minimal tissue removal or to perform a more extensive re-excision or even escalate to a total mastectomy. However, this decision-making process is challenging due to the paucity of information regarding the risk of secondary margin positivity.

Our predictive model addresses this gap by integrating preoperative and intraoperative data available at the decision point to provide critical information about the risk of secondary margin positivity in subsequent re-excisions. This information allows surgeons to make more informed decisions, potentially reducing the likelihood of persistently positive margins. For example, in cases that are considered low risk (risk score below 97 points), surgeons can confidently opt for more conservative re-excisions, preserving healthy breast tissue and potentially improving cosmetic outcomes without compromising oncologic safety. Conversely, in high-risk cases (risk score above 97 points), surgeons may consider more extensive re-excision or even total mastectomy to minimize the likelihood of persistently positive margins and the need for further re-excision.

The implementation of this predictive nomogram in clinical practice has the potential to provide multiple benefits across multiple aspects of breast cancer surgery. By facilitating more informed decision-making during the critical intraoperative period following an initial positive margin, this model could contribute to a reduction in operative time. This temporal efficiency is particularly valuable in the context of breast-conserving surgery, where prolonged anesthesia time may increase patient risk. In addition, the model’s ability to stratify risk for secondary margin positivity could lead to a reduction in unnecessary re-excisions, thereby reducing pathologist workload. This is particularly important given the time-sensitive nature of frozen section analysis and the potential for resource constraints in pathology departments. From a patient-centered perspective, the use of the model may result in a reduction in surgical risks associated with prolonged surgery or multiple re-excisions. In addition, by potentially reducing the need for repeat surgeries, the model could contribute to an overall reduction in the cost of breast cancer care. Taken together, these factors result in an improved surgical decision-making process that enables more precise and personalized care. This alignment with the principles of patient-centered care represents a significant advancement in the field of surgical oncology, potentially improving both clinical outcomes and patient satisfaction.

This study represents a significant advancement in the application of predictive modeling for margin status in BCS. Previous research in this area has primarily focused on developing models based on preoperative paraffin pathology and/or immunohistochemistry data (15–17). In addition, these earlier models were generally designed to predict initial margin positivity (16, 18–20). However, such approaches have limited applicability in surgical settings where FSA is routinely used intraoperatively to determine tumor type and assess margins. In these scenarios, preoperative pathological and immunohistochemical information about the tumor is often not available. Furthermore, existing models are not adapted to predict the risk of secondary margin positivity in cases where re-excision and subsequent FSA are performed during the same surgical session following an initial positive margin. Our study addresses this critical gap by developing a nomogram specifically tailored to this surgical workflow. To our knowledge, this is the first model applicable to such a surgical workflow in BCS. By utilizing both preoperative and intraoperative data, including initial FSA results, our model provides a novel tool for real-time risk stratification of secondary margin positivity. This extension of predictive modeling to intraoperative decision making represents a significant step forward in the field of surgical oncology, potentially improving the accuracy and efficiency of BCS procedures.

The model demonstrates robust performance across multiple statistical measures. Its discriminative ability is evidenced by mean area under the curve (AUC) values of 0.79 (95% CI: 0.72-0.85) in the training set and 0.83 (95% CI: 0.71-0.92) in the test set, indicating strong predictive accuracy. Good calibration is supported by good agreement between predicted and actual outcomes, as demonstrated by calibration curves and Hosmer-Lemeshow test p-values of 0.214 and 0.167 in the training and test sets, respectively. The clinical utility of the model is demonstrated by DCA, which showed consistent superiority over “treat all” and “treat none” strategies. At the optimal threshold of 97 points on the nomogram, the model achieved balanced sensitivity and specificity in both sets (training set: 0.66 and 0.80; test set: 0.65 and 0.88, respectively). These consistent results across training and test sets, supported by bootstrap resampling for confidence interval estimation, provide compelling evidence of the model’s reliability and potential clinical value in identifying patients at high risk for secondary margin positivity during BCS procedures.

Our study found a significant correlation between invasive lobular carcinoma (ILC) and secondary margin positivity (*p* < 0.05). This association confirms previous findings that ILC is associated with an increased risk of initial positive margins in BCS (42–46). The peculiar biology of ILC, in particular the loss of E-cadherin leading to a discohesive cell structure, makes it difficult to achieve clear surgical margins (47). In addition, our study uncovers a novel finding: the correlation between ultrasound margins with indistinct microlobulated features and secondary margin positivity (*p* < 0.05). This novel observation highlights the need for further investigation. Furthermore, we found a potential but not statistically significant correlation between obscured mammographic margins and secondary margin positivity. Typically, obscured mammographic margins, which are often associated with dense breast tissue, can compromise the accuracy of mammographic imaging (48) and challenge the accurate localization and sizing of tumors (49). Our results highlight the critical role of considering the masking effect of dense breast tissue in preoperative planning. This suggests that patients with dense breasts may benefit from additional caution or alternative imaging modalities to reduce the risk of positive surgical margins.

Our study has several limitations:

1. Retrospective nature: As a retrospective study, there is an inherent potential for selection bias. For example, some patients with positive initial margins may have undergone total mastectomy or refused further re-excision. This study excludes their data from the analysis of secondary margin status, thus introducing selection bias. However, the number of such patients in our study was relatively small, mitigating the effect of this bias.

2. The relatively small sample size of our study poses a potential risk of model overfitting. To minimize this problem, we implemented LASSO regression to reduce the likelihood of overfitting in model construction. In addition, we used the bootstrap method to increase the accuracy of assessing model performance with a reduced bias in model validation. However, despite these precautions, the risk of overfitting and overestimation remains due to the small sample size. This concern calls for a cautious interpretation of our results. The limitation of the small sample size highlights the need for future research with larger and more diverse datasets.

3. Lack of external validation: This study did not include an external validation set from other medical institutions. Although we attempted to reduce assessment bias by dividing our dataset into training and testing sets based on different admission periods, the model’s performance in different clinical settings is still limited due to the lack of external validation. Thus, the nomogram requires validation in multiple clinical settings before it can be integrated into clinical practice.

4. This study is based on the assumption that surgeons can effectively intervene in cases where patients are identified as high risk to prevent the occurrence of secondary positive margins. However, the actual effectiveness of the model in reducing the incidence of secondary positive margins in real-world surgical settings has yet to be empirically validated. This underlying assumption is critical to the application of the model and underscores the need for further, more comprehensive investigation. Future research should focus on standardized preventive measures and rigorous clinical validation of the model’s effectiveness in reducing secondary positive margins. The implementation of randomized controlled trials is particularly important, as they would provide robust and definitive evidence of the utility and efficacy of the model in clinical practice.

## 5 Conclusion

In conclusion, this study presents an innovative nomogram for predicting secondary margin positivity in BCS with FSA-assisted excisional biopsy and margin assessment. This predictive tool addresses a critical gap in existing models by seamlessly integrating preoperative clinical data with intraoperative pathology findings, enabling real-time risk stratification at a critical decision point during surgery.

Implementation of this nomogram could have multiple benefits, including reduced operative times, reduced pathologist workload, and reduced surgical risks and costs for patients. The model’s potential to optimize surgical planning, reduce re-excision rates, and improve resource utilization represents a significant advancement toward more personalized and efficient breast cancer care.

However, we recognize that further validation and research is essential. Future investigations should prioritize external validation through multi-center studies, and cost-effectiveness analyses will help quantify the economic benefits of implementation of this predictive tool in clinical practice. Furthermore, prospective studies evaluating the practical integration of the nomogram into existing clinical workflows will be essential to ensure its seamless adoption and maximize its utility in real-world surgical scenarios.

While these additional steps are necessary before advocating for widespread clinical adoption, this nomogram offers a promising avenue for improving decision making in breast cancer surgery. By providing surgeons with more accurate, patient-specific risk information, it has the potential to significantly improve both clinical outcomes and patient satisfaction in breast cancer management. This tool represents a tangible step toward the realization of personalized medicine in surgical oncology, potentially changing the landscape of breast cancer treatment and setting a new standard for intraoperative decision-making tools in cancer care.

## Data Availability

The raw data supporting the conclusions of this article will be made available by the authors, without undue reservation.
